# Optimizing Sensitivity in a Fluid-Structure Interaction-Based Microfluidic Viscometer: A Multiphysics Simulation Study

**DOI:** 10.3390/s23229265

**Published:** 2023-11-18

**Authors:** Adil Mustafa, Merve Ertas Uslu, Melikhan Tanyeri

**Affiliations:** 1 Department of Engineering Mathematics, University of Bristol, Bristol BS8 1TW, UK; adil.mustafa@bristol.ac.uk; 2 Department of Biomedical Engineering, School of Science and Engineering, Duquesne University, Pittsburgh, PA 15282, USA; ertasuslum@duq.edu

**Keywords:** microfluidic viscometer, fluid-structure interaction, micropillar, deflection, multiphysics simulations

## Abstract

Fluid-structure interactions (FSI) are used in a variety of sensors based on micro- and nanotechnology to detect and measure changes in pressure, flow, and viscosity of fluids. These sensors typically consist of a flexible structure that deforms in response to the fluid flow and generates an electrical, optical, or mechanical signal that can be measured. FSI-based sensors have recently been utilized in applications such as biomedical devices, environmental monitoring, and aerospace engineering, where the accurate measurement of fluid properties is critical to ensure performance and safety. In this work, multiphysics models are employed to identify and study parameters that affect the performance of an FSI-based microfluidic viscometer that measures the viscosity of Newtonian and non-Newtonian fluids using the deflection of flexible micropillars. Specifically, we studied the impact of geometric parameters such as pillar diameter and height, aspect ratio of the pillars, pillar spacing, and the distance between the pillars and the channel walls. Our study provides design guidelines to adjust the sensitivity of the viscometer toward specific applications. Overall, this highly sensitive microfluidic sensor can be integrated into complex systems and provide real-time monitoring of fluid viscosity.

## 1. Introduction

In recent years, the integration of microfluidic techniques has revolutionized numerous fields of science and technology, offering precise and controlled manipulation of fluid samples [1,2,3,4]. Microfluidic techniques present a myriad of advantages including the ability to handle small sample volumes [5], achieving high precision [6], enabling real-time monitoring [7,8], and multiplexing [9], with a wide range of potential applications in biomedical [10,11,12,13], pharmaceutical [14,15], food science and engineering [16], and petroleum [17] industries. Viscosity is a fundamental property of fluids, playing a critical role in a diverse scientific and industrial applications. Accurate viscosity measurements are essential for optimizing processes and understanding fluid behavior in complex systems. For example, measuring the viscosity of biofluids such as blood, saliva, and mucus provides valuable information for disease diagnosis, monitoring therapeutic treatments, and predicting biological functions [18].

Microfluidics has emerged as a powerful tool for viscosity determination, offering unique advantages due to its ability to handle small fluid volumes, typically in the microliter or nanoliter range, with high precision and throughput. Microfluidic viscometry techniques encompass various principles of measurement, including pressure and flow rate sensing, surface tension, co-flow schemes, and droplet-based methods [19,20]. For instance, pressure sensing viscometers measure pressure drop across microchannels to calculate viscosity, while surface tension viscometers rely on capillary pressure and wetting properties. Microfluidic techniques enable viscosity measurement in various fluids, including non-Newtonian and complex fluids, with particular relevance to biomedical applications where many biological fluids exhibit non-Newtonian behavior.

We have recently developed a novel method for measuring the viscosity of Newtonian and non-Newtonian fluids [21,22]. This method involves fluid-structure interactions and measures the deflection of elastic polydimethylsiloxane (PDMS) micropillars in response to fluid flow to determine the viscosity. In this method, fluids are introduced to the microchannel containing a flexible micropillar array at various flow rates to image and analyze the micropillar deflection caused by fluid flow. By measuring the position of the micropillar tip both under static conditions (no flow) and under fluid flow, we determine the net micropillar tip displacement along the flow direction, thereby establishing a direct correlation with viscosity. Using this method, we previously demonstrated viscosity measurements of Newtonian fluids with a sensitivity and dynamic range of 0.5 cP and 2–100 cP, respectively.

While our method inherits several advantages common to microfluidic viscometry, including small sample volume and adaptability to complex fluids, it distinguishes itself by relying on fluid-structure interactions, obviating the need for integrated pressure or flow sensors within the device design. Nevertheless, a notable limitation arises from the necessity to establish a calibration curve that correlates micropillar deformation with fluid viscosity. To address this limitation and to better understand the relationship between the fluid viscosity and micropillar deformation, we decided to explore the parameters that influence the sensitivity of our viscometer.

In this study, we investigated the parameters impacting the sensitivity of our microfluidic viscometer. Given that the viscometer primarily relies on fluid-structure interactions, we chose to focus on key parameters of device geometry that directly affect the deflection of micropillars induced by fluid flow. These parameters include micropillar dimensions, micropillar aspect ratio, pillar spacing, the distance between the micropillars and the channel walls, and Young’s modulus of the micropillars. We employed multiphysics simulations to examine how changes in these parameters impact the sensitivity of the viscometer. These simulations are based on multiphysics models that we developed and experimentally validated in our previous studies [21,22] (also see Section S2 in Supplementary Materials). Our results will help advance the application of this microfluidic viscometer to a broader range of fluids and facilitate custom viscometer design towards specific applications.

## 2. Device Design

**Figure 1:**
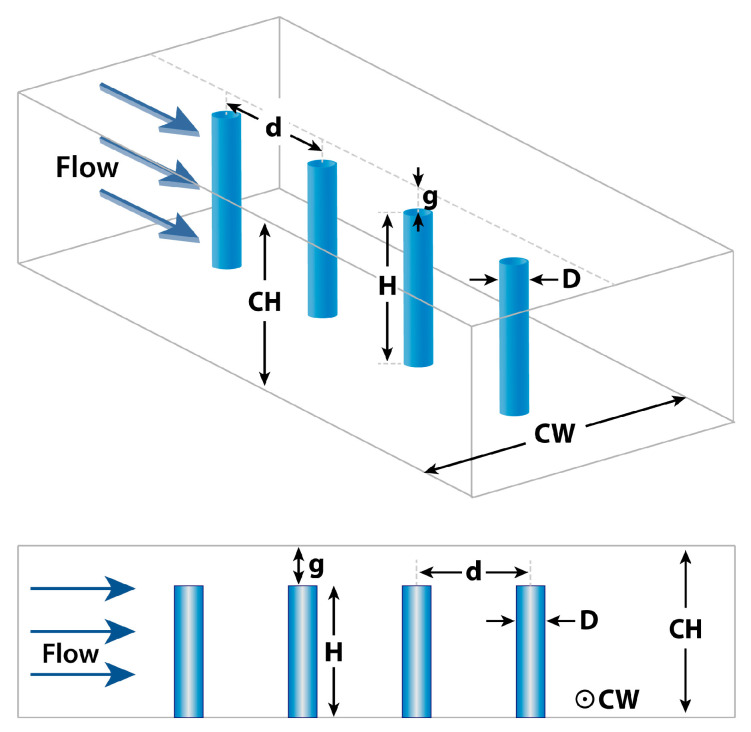
Geometric design parameters utilized in the FSI-based microfluidic viscometer. D: pillar diameter, H: pillar height, g: gap between micropillar tip and channel ceiling, d: pillar spacing, CW: channel width, CH: channel height.

The microfluidic viscometer utilized in the simulations comprises a rectangular microchannel with a single array of 10 micropillars. We opted for a rectangular channel geometry due to its compatibility with standard fabrication methods for micropillars. Figure 1 illustrates a subset of these micropillars and presents the geometric parameters examined in this study to assess their impact on device performance. Micropillar diameter and height are denoted by D and H, respectively. The distance between two neighboring micropillars is represented by d, and the gap between the micropillar tip and the channel ceiling is denoted by g. The width and height of the microchannel are represented by CW and CH, respectively. The aspect ratio (AR) of the micropillars is defined as AR=H/D. For clarity and consistency throughout the manuscript, we will henceforth refer to these geometric parameters by their assigned alphabet letters.

## 3. Materials and Methods

### 3.1. Multiphysics Modelling

Our multiphysics model for the microfluidic viscometer device was based on fluid-structure interactions by coupling the laminar form of the Navier–Stokes equation to strain equations for linear elastic polymers. The momentum equations used to model fluid flow are given as [23]:

(1) $$\rho \left(u\cdot \nabla \right)u=\nabla \cdot \left[-pI+\mu \left(\nabla u+{\left(\nabla u\right)}^{T}\right)\right]+F$$

where ρ, μ, and u denote the fluid density, viscosity, and velocity, respectively. p is the pressure and F is the volume force. The fluid was modelled as an incompressible fluid by coupling Equation (1) with the continuity equation:

(2) ∇·u=0

Equations of motion for the strains were solved to account for the displacement of PDMS micropillars under shear stress using [24]:

(3) $$0=F_{V}-\nabla_{X}P^{T}$$

where $F_{V}$ is the volume force vector, and P is the first Piola–Kirchhoff stress tensor. The first Piola–Kirchhoff stress P (or nominal stress) is calculated by P=FS, where F is the deformation gradient and S is the second Piola–Kirchhoff stress. The COMSOL Multiphysics fluid-structure interaction (FSI) interface was used to couple fluid flow with solid mechanics to achieve the desired fluid-structure interaction. FSI couplings appear on the boundaries between the fluid and the solid. The interface uses an arbitrary Lagrangian–Eulerian (ALE) method to combine the fluid flow with solid mechanics. A user-controlled mesh was created with a minimum and maximum element size of 126 and 700 µm, respectively. Entire geometry was calibrated for fluid dynamics using a free tetrahedral subnode which in turn creates an unstructured mesh. Boundary layer properties were adjusted by choosing a boundary layer stretching factor of 1.2 and a thickness adjustment factor of 5. It was assumed that field variables do not change over time, and therefore a stationary study node was used for computations. Details on simulation methodology and parameters are provided in Supplementary Materials Section S1.

### 3.2. Simulation Parameters

The important simulation parameters for the microfluidic viscometer include the range of viscosities and flow rates as well as the micropillar and channel dimensions. Following the design parameters utilized in our previous experimental work [21,22], we examined a viscosity range of *μ* = 5–100 cP, and a flow rate between Q = 15–105 mL/h. For the micropillar and channel dimensions, we set the diameter of the micropillars at D=300 µm, and varied the pillar aspect ratio (AR) between 3 and 5, corresponding to a pillar height between H=900 and 1500 µm. Further, we varied g (the gap between the micropillar tip and the channel ceiling) between 50 and 300 µm, and d (pillar spacing) between 350 and 600 µm. The channel width (CW) was varied between 700 and 900 µm, implying a gap of 400–600 μm between the pillar and the channel side walls. Finally, the Young’s modulus was varied between 1.3 and 3.1 MPa. We assumed that the initial device parameters were: D=300 µm and H=1500 µm, corresponding to a micropillar aspect ratio of 5:1, g=100 µm, d=400 µm, and CW=900 µm.

We investigated the pillar displacement from all 10 micropillars. We observed that the micropillars in the middle of the array (Pillar #4, #5, and #6) displayed maximum displacement with high consistency. As a result, we picked Pillar #5 to study the impact of micropillar and channel dimensions on the viscometer performance.

## 4. Results and Discussion

The sensitivity of our microfluidic viscometer device depends on the geometric parameters of the micropillars and the channel. To investigate the impact of these parameters on the viscometer performance, we systematically modified each parameter and carried out simulations. Below, we provide the results from each study.

### 4.1. Pillar Aspect Ratio (AR)

We initially investigated the influence of pillar aspect ratio on microfluidic viscometer performance. To this end, we analyzed the displacement of pillars for micropillar arrays characterized by three aspect ratios: 3:1, 4:1, and 5:1. While maintaining a constant micropillar diameter (D=300 µm), we varied the micropillar height (H=900,1200,and 1500 µm) for each aspect ratio. For the micropillar array with an aspect ratio of AR=5:1 (D=300 µm, H=1500 µm), the flow rate is varied between 15 and 105 mL/h. Meanwhile, for AR=4:1 and AR=3:1, we adjusted the channel height (CH) to maintain a constant gap (g=100 µm) between the micropillar tip and the channel ceiling. Consequently, for AR=3:1, AR=4:1, and AR=5:1, the channel heights were adjusted to ${CH}_{AR=3:1}=1000\;µm$, ${CH}_{AR=4:1}=1300\;µm$, and ${CH}_{AR=5:1}=1600\;µm$, respectively. This necessitated adjusting the flow rates to obtain equal average flow velocities across the microchannel, following the equation:

(4) $$Q_{A}=Q_{5:1}\times \frac{{CH}^{\prime }}{{CH}_{5:1}}$$

where $Q_{A}$ is the adjusted flow rate, $Q_{5:1}$ and ${CH}_{5:1}$ are the flow rates and the channel height for the viscometer with AR=5:1, and ${CH}^{\prime }$ is the channel height (${CH}_{4:1}$ or ${CH}_{3:1}$) for the viscometer with either AR=4:1 or AR=3:1. For instance, a $Q_{5:1}=15\;mL/h$ would correspond to $Q_{4:1}=Q_{5:1}\times {CH}_{4:1}/{CH}_{5:1}=15\times 1300/1600=12.19\;mL/h,$ and $Q_{3:1}=Q_{5:1}\times {CH}_{3:1}/{CH}_{5:1}=15\times 1000/1600=9.38\;mL/h$.

**Figure 2:**
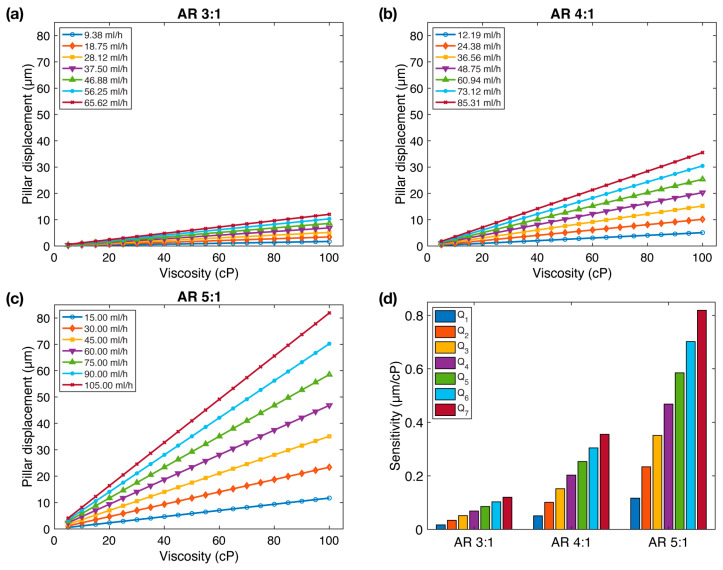
The impact of aspect ratio on FSI-based microfluidic viscometer sensitivity: (**a**–**c**) Micropillar displacement as a function of fluid viscosity at various flow rates for three different micropillar aspect ratios. (**d**) Sensitivity (s) of the viscometer as a function of aspect ratio. The sensitivity of the viscometer increases with aspect ratio.

Figure 2 illustrates the pillar displacement as a function of viscosity for all three aspect ratios. As anticipated, we observed a linear relationship between pillar displacement, flow rate, and viscosity. To assess the microfluidic viscometer’s performance, we introduced a parameter labeled as s, representing the slope of the pillar displacement versus viscosity curves. This parameter s serves as an indicator of sensitivity, reflecting the pillar displacement per unit viscosity change. Greater sensitivity corresponds to higher pillar displacement per unit viscosity change, indicating a more responsive viscometer.

We observe that the sensitivity, s, demonstrates a remarkable increase with aspect ratio (Figure 2d). Notably, while s ranges between 0.0172 and 0.1204 µm/cP for AR=3:1, this range expands to 0.0507– 0.3552 µm/cP for AR=4:1, and 0.1170–0.8192 µm/cP for AR=5:1, representing a 2.95× and 6.81× increase in sensitivity, respectively.

### 4.2. Gap between the Pillar Tip and Channel Ceiling (g)

**Figure 3:**
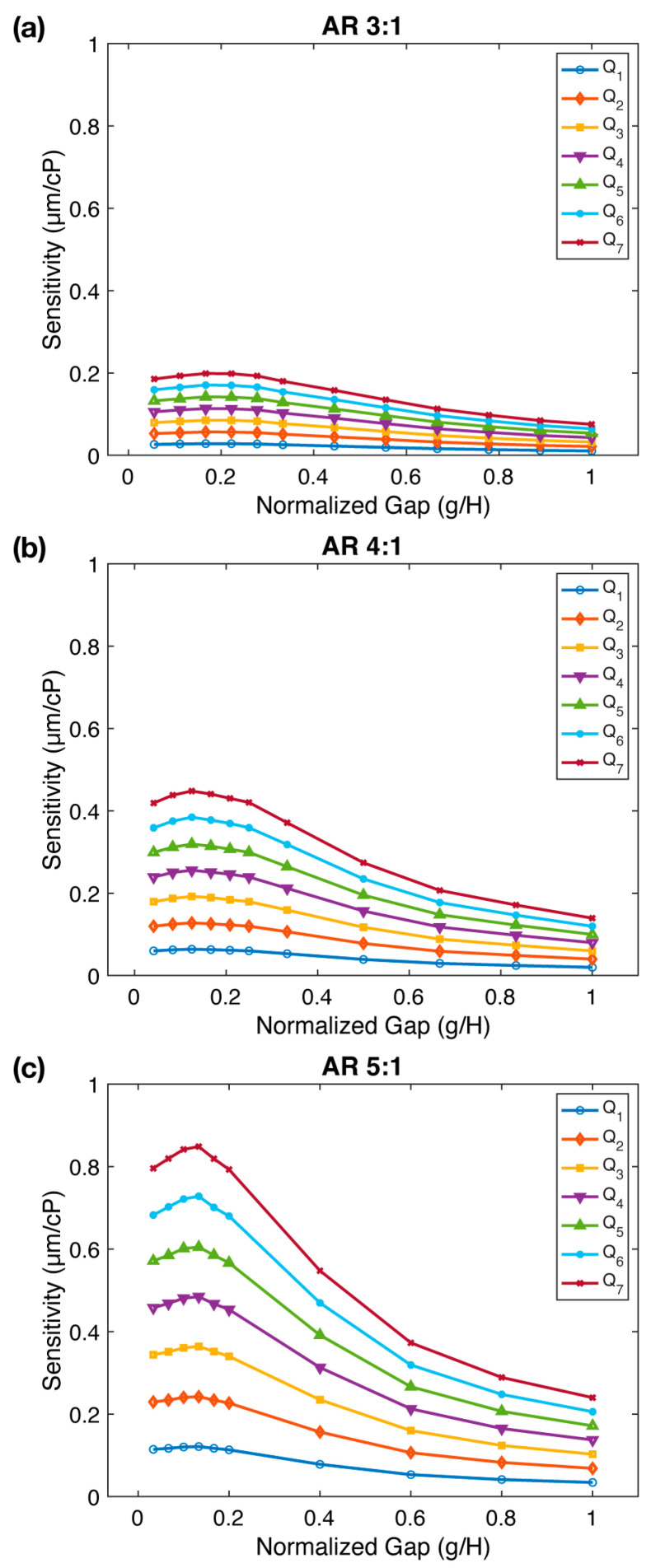
The impact of gap (g) between the micropillar tip and channel ceiling on FSI-based microfluidic viscometer sensitivity. The sensitivity (s) of the viscometer as a function of the normalized gap (g/H) for three different micropillar aspect ratios: (**a**) AR=3:1, (**b**) AR=4:1, and (**c**) AR=5:1. The sensitivity reaches a maximum at normalized gap values of g/H=0.1667, g/H=0.125, and g/H=0.1333 for AR=3:1, AR=4:1, and AR=5:1, respectively. Flow rates ($Q_{1}-Q_{7})$ are provided in Figures S3–S5.

We then investigated the influence of the gap between the pillar tip and channel ceiling, denoted as g, on microfluidic viscometer performance. We anticipated that as we increase g, the fluid-micropillar interaction would decrease due to reduced fluidic resistance arising from the widened gap (Figure S5a,b). We studied the impact of g for all three aspect ratios as depicted in Figure 3 and Figures S3–S5. Throughout the simulations, the micropillar diameter (D=300 µm) was kept constant, and we systematically varied the gap size from g=50 µm up to g=H (micropillar height) for each aspect ratio. To accommodate the varying gap sizes, corresponding adjustments were made to the channel height (CH), extending it up to twice the height of the micropillar (CH=2×H). This ensured that the micropillar tip would be situated at the middle of the channel height when the gap was at its maximum value. The channel height (CH) was adjusted to the following values for the respective aspect ratios: ${CH}_{3:1}=950–1800\;µm$, ${CH}_{4:1}=1250–2400\;µm$, and ${CH}_{5:1}=1550–3000\;µm$. To attain equal average flow velocities across the microchannel for all aspect ratios, the flow rate was modified following Equation (5):

(5) $$Q_{A}=Q_{g=100}\times \frac{{CH}^{\prime }}{{CH}_{g=100}}$$

where $Q_{A}$ represents the adjusted flow rate, $Q_{g=100}$ and ${CH}_{g=100}$ are the initial flow rate and channel height at g=100 µm, and ${CH}^{\prime }$ is the adjusted channel height. For instance, for micropillars with AR=3:1, increasing the gap to g=300 µm would entail adjusting an initial flow rate of Q=15 mL/h to $Q_{A}=Q\times \left({CH}^{\prime }/CH\right)=15\times \left(1200/1000\right)=18\;mL/h$. Please refer to the figure legends in Figures S3–S5 for specific flow rates under each condition. Our investigation encompassed flow rates spanning from 15 to 105 mL/h for g=100 µm at each aspect ratio, with appropriate flow rate adjustments for other gap values as indicated in Figures S3–S5 legends.

Figure 3 illustrates the relationship between sensitivity (s) and gap values normalized with respect to the micropillar height (g/H) for all three aspect ratios. We observe that an increase in the gap results in a reduction in sensitivity; however, intriguingly, it attains a maximum value before this decline. This pattern prevails across all aspect ratios, with sensitivity slightly enhancing in the range of g/H=0.125–0.166 followed by a subsequent decline with further gap expansion. As the gap is increased to g/H=1, the sensitivity drops by a factor of 2.64×, 3.21×, and 3.54× from its peak value for AR=3:1, AR=4:1, and AR=5:1, respectively. Sensitivity measurements at three different aspect ratios confirmed our prior observation that sensitivity increases significantly with aspect ratio. In summary, we conclude that while an increase in the gap diminishes sensitivity, there exists an optimal gap value that maximizes the viscometer sensitivity. This is likely because, as the normalized gap increases from very small values (e.g., g=50 µm or g/H=0.03–0.05) to g=150–200 µm or g/H=0.125–0.166, sensitivity slightly increases due to increased fluid-structure interaction. However, at higher g/H ratios, the gap is expanded, allowing the fluid to escape through the gap, effectively diminishing the fluid-structure interaction and resulting in reduced sensitivity.

### 4.3. Channel Width (CW)

**Figure 4:**
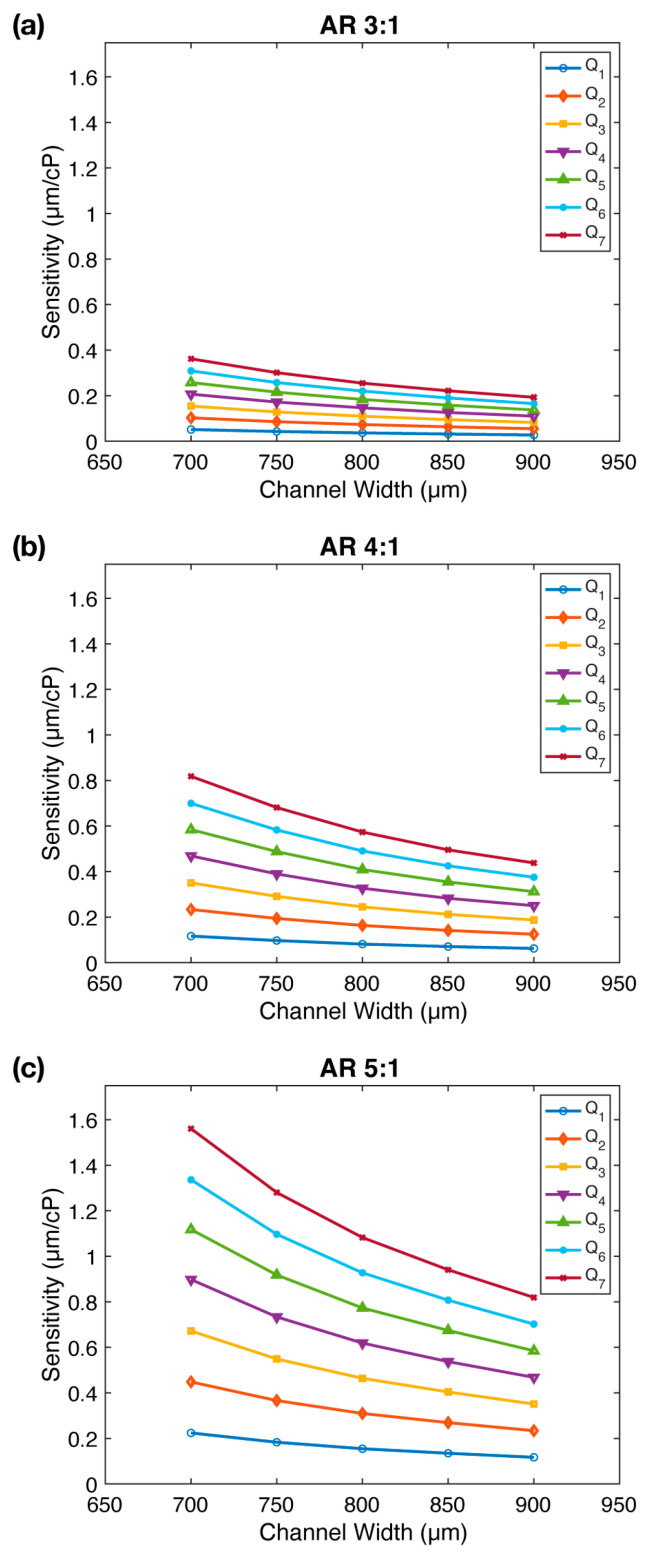
The impact of channel width (CW) on FSI-based microfluidic viscometer sensitivity: (**a**–**c**) The sensitivity (s) of the viscometer as a function of the channel width (CW) for three different micropillar aspect ratios. The sensitivity increases with decreasing channel width for all aspect ratios. Flow rates ($Q_{1}-Q_{7})$ are provided in Figures S6–S8.

Subsequently, we investigated the influence of the channel width (CW) on the sensitivity, or the gap between the micropillars and the channel side walls. Our expectation was that a decrease in CW would promote fluid-micropillar interactions due to higher fluidic resistance arising from a narrower microchannel cross-sectional area. We studied the impact of CW for all three aspect ratios as depicted in Figure 4 and Figures S6–S8. While maintaining a constant micropillar diameter (D=300 µm), we systematically varied the channel width between CW=700 and 900 µm for each aspect ratio. With a pillar diameter of D=300 µm, this would leave a gap of 400–600 µm between the micropillar and the microchannel side walls. For the micropillar array with CW=900 µm, flow rates spanned from 15 to 105 mL/h (Figures S6–S8). For other channel widths, the flow rate was modified following Equation (6) to attain equal average flow velocities across the microchannel:

(6) $$Q_{A}=Q\times \frac{{CW}^{\prime }}{CW}$$

where $Q_{A}$ is the adjusted flow rate, Q is the flow rate at CW=900 µm, and ${CW}^{\prime }$ is the adjusted channel width. For instance, when the channel width is reduced to ${CW}^{\prime }=700\;µm,$ an initial flow rate of Q=15 mL/h would be adjusted to $Q_{A}=Q\times \left({CW}^{\prime }/CW\right)=15\times \left(700/900\right)=11.67\;mL/h$. Please refer to the figure legends in Figures S6–S8 for specific flow rates under each condition.

Figure 4 demonstrates the correlation between sensitivity (s) and channel width (CW) for all three aspect ratios. Intriguingly, a decrease in channel width leads to enhanced sensitivity, with an average improvement of 1.87×, 1.87×, and 1.91× for AR=3:1, AR=4:1, and AR=5:1, respectively. Notably, this increase in sensitivity is most pronounced within the mid-range flow rates. Moreover, upon comparing panels in Figure 4, we reaffirm the earlier observation that sensitivity increases substantially with aspect ratio. We conclude that decreasing the channel width (CW) increases the viscometer sensitivity.

### 4.4. Pillar Spacing (d)

**Figure 5:**
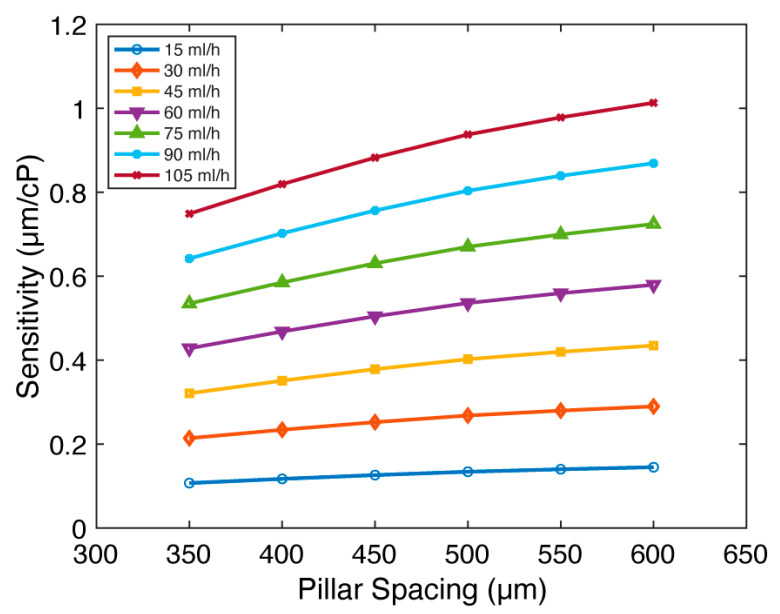
The impact of pillar spacing (d) on FSI-based microfluidic viscometer sensitivity. The sensitivity (s) of the viscometer as a function of the pillar spacing (d) for flow rates between 15 and 105 mL/h. The sensitivity moderately increases with pillar spacing.

In addition, we investigated the effect of pillar spacing (d), which is defined as the center-to-center distance between micropillars. We postulated that increasing the pillar spacing could mitigate the “shielding” effect arising from the arrangement of the micropillars in a single line along the direction of fluid flow. Such an increase in pillar spacing was anticipated to increase fluid-micropillar interactions, leading to increased sensitivity. We examined the impact of pillar spacing for micropillars with AR=5:1 (Figure 5 and Figure S9). While keeping all other parameters constant (D=300 µm, H=1500 µm, g=100 µm, and CW=900 µm), we varied the pillar spacing across d=350–600 µm, equivalent to 50–300 µm of surface-to-surface distance between micropillars. Flow rates are set between 15 and 105 mL/h for all pillar spacings. Figure 5 presents the relationship between sensitivity (s) and pillar spacing (d). Confirming our hypothesis, we observed that the sensitivity increases with pillar spacing. Indeed, a consistent sensitivity improvement of 1.35× is obtained at each flow rate as the pillar spacing is increased from d=350 µm to d=600 µm. We conclude that the viscometer sensitivity moderately increases with the expansion of pillar spacing.

### 4.5. Young’s Modulus (E)

**Figure 6:**
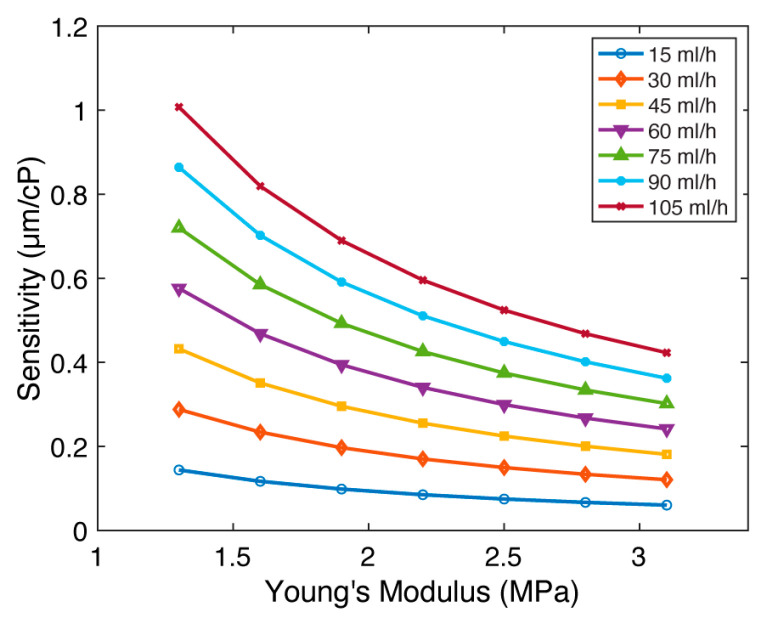
The impact of Young’s modulus (E) on FSI-based microfluidic viscometer sensitivity. The sensitivity (s) of the viscometer as a function of the Young’s modulus (E) for flow rates between 15 and 105 mL/h. The sensitivity decreases with Young’s modulus.

Finally, we investigated the impact of Young’s modulus (E) on micropillar sensitivity. Young’s modulus is a measure of pillar stiffness, and its increase results in a more rigid structure. We hypothesized that this enhanced rigidity would compromise the micropillar’s bending capacity, leading to a decline in sensitivity. Our investigation focused on micropillars with an aspect ratio of 5:1 (Figure 6 and Figure S10). While keeping all other parameters constant (D=300 µm, H=1500 µm, g=100 µm, and CW=900 µm), we varied Young’s modulus across the range of E=1.3–3.1 MPa, reflecting established values for polydimethylsiloxane (PDMS) [25,26]. Flow rates were set between 15 and 105 mL/h for all Young’s modulus values. Figure 6 illustrates the relationship between sensitivity (s) and Young’s modulus (E). The results demonstrate that sensitivity diminishes as Young’s modulus increases, reflecting a substantial sensitivity reduction of 2.38× at each flow rate. We conclude that the viscometer sensitivity decreases with increased Young’s modulus.

## 5. Conclusions

In summary, we utilized multiphysics simulations to investigate the influence of device geometry on the sensitivity of our FSI-based microfluidic viscometer. Notably, the aspect ratio of the micropillars emerged as a pivotal factor in sensitivity enhancement. We observed a remarkable 6.81× increase in sensitivity as the aspect ratio was raised from 3:1 to 5:1. Intriguingly, there is an optimal gap between the micropillar tip and channel ceiling that maximizes the sensitivity. This optimal gap value, when normalized against the micropillar height, falls within the range of g/H=0.125–0.166. Beyond this range, the sensitivity drops sharply, up to 3.54×, as the gap is increased to a full micropillar height (g/H=1). Conversely, a reduction of 22% in the channel width led to a notable improvement of approximately 1.9× in sensitivity, a trend consistently observed across all aspect ratios and particularly pronounced at mid-range flow rates (e.g., 60–75 mL/h). Additionally, widening the pillar spacing resulted in improved sensitivity, contributing to an average 1.35× enhancement across all flow rates. Finally, increasing the Young’s modulus by 2.38× resulted in a corresponding decrease in the sensitivity by the same amount. Based on these findings, we conclude that the parameters affecting viscometer sensitivity, in order of importance, are aspect ratio (AR), gap between the pillar tip and the channel ceiling (g), Young’s modulus (E), channel width (CW), and pillar spacing (d). These findings collectively underscore the influence of key geometric parameters on the sensitivity of the microfluidic viscometer, offering critical insights for tailored device design and optimized performance across diverse applications.

For designing a micropillar-based viscometer with high sensitivity, we recommend the following guidelines based on our findings:

1. Aspect Ratio Enhancement: Our study demonstrated a substantial increase in sensitivity with aspect ratio. Consider employing micropillars with aspect ratios of 4:1 or higher, as these configurations exhibited notable sensitivity gains.
2. Optimal gap between the micropillar tip and the channel ceiling: Maintaining a gap-to-pillar height ratio within g/H=0.125–0.166 not only maximizes sensitivity, but also ensures an accommodating gap size for facile and consistent device fabrication.
3. Young’s modulus: While a low Young’s modulus enhances sensitivity, it is essential to consider the structural integrity of the micropillars and potential fabrication challenges when dealing with excessively low values.
4. Channel Width Reduction: Decreasing the channel width enhances the sensitivity of the viscometer. Narrowing the cross-sectional area of the microchannel intensifies fluid-micropillar interactions.
5. Pillar Spacing Expansion: Increasing the space between micropillars mitigates the shielding effect, fostering stronger fluid-micropillar interactions. Our investigation revealed a consistent sensitivity enhancement with increased pillar spacing.

On the other hand, enhancing the sensitivity of a micropillar based viscometer can lead to a trade-off with its dynamic range, the range of viscosity values it can effectively measure. Amplifying sensitivity leads to a larger micropillar displacement for every unit change in viscosity. Consequently, for a given device geometry, the maximum pillar displacement could be achieved within a smaller range of viscosity, thereby curtailing the dynamic range of measurements. Achieving a balance between sensitivity and dynamic range depends on the unique requirements of the application and should be carefully considered during the design phase. When high sensitivity is crucial and expected viscosity variations are within a relatively narrow range, designing the micropillar viscometer for maximum sensitivity may be suitable. Conversely, for applications involving a wide range of viscosities, achieving a wider dynamic range may come at the expense of sensitivity. One potential strategy to mitigate this trade-off is to incorporate multiple micropillars with varying geometries, such as micropillar dimensions and aspect ratios, within the viscometer. This approach allows for the extension of the dynamic range without substantially compromising the sensitivity.

While this study was focused on the behavior and sensitivity of the viscometer under steady laminar flow conditions, it is worth noting that our viscometer has the potential to deduce rheological properties of fluids during transient states [27,28], which could be valuable for specific applications.

Understanding how geometric parameters influence viscometer sensitivity lays the framework for designing microfluidic viscometers tailored to specific real-world applications. Such tailored viscometers hold the potential to enhance medical diagnostics by ensuring more accurate tests (e.g., blood coagulation tests), improve industrial processes by optimizing them for specific fluids, and bolster reliability in environmental monitoring, particularly in complex fluid environments such as wastewater. This foundational knowledge opens avenues for developing microfluidic viscometers capable of handling diverse and dynamic fluid conditions, prompting further exploration of their performance in scenarios involving varying fluid viscosities, temperatures, and flow rates.

Optimizing performance parameters, such as sensitivity and dynamic range, of the FSI-based microfluidic viscometer for a specific application is crucial to ensure versatility across a wide spectrum of potential applications, including medical diagnostics, industrial processes, and environmental monitoring.

## Data Availability

The data presented in this study are available upon request from the corresponding author.

## Supplementary Materials

The supporting information can be downloaded at: https://www.mdpi.com/article/10.3390/s23229265/s1. Figure S1: title; Table S1: title; Video S1: title. Table S1: Simulation Methodology; Figure S1: Impact of mesh size on simulation results; Figure S2: Experimental validation of the multiphysics model for the microfluidic viscometer; Figures S3-S5: Investigating the impact of gap (g) on viscometer sensitivity; Figure S6: Investigating the impact of channel width (CW) on viscometer sensitivity for micropillars with AR=3:1; Figure S7: Investigating the impact of channel width (CW) on viscometer sensitivity for micropillars with AR=4:1; Figure S8: Investigating the impact of channel width (CW) on viscometer sensitivity for micropillars with AR=5:1; Figure S9: Investigating the impact of pillar spacing (d) on viscometer sensitivity for micropillars with AR=5:1; Figure S10: Investigating the impact of Young’s modulus (E) on viscometer sensitivity for micropillars with AR=5:1.

## References

[1] Nunes J.K., Stone H.A. (2022). Introduction: Microfluidics. Chem. Rev..

[2] Preetam S., Nahak B.K., Patra S., Toncu D.C., Park S., Syväjärvi M., Orive G., Tiwari A. (2022). Emergence of microfluidics for next generation biomedical devices. Biosens. Bioelectron. X.

[3] Pandey C.M., Augustine S., Kumar S., Kumar S., Nara S., Srivastava S., Malhotra B.D. (2018). Microfluidics Based Point-of-Care Diagnostics. Biotechnol. J..

[4] Beebe D.J., Mensing G.A., Walker G.M. (2002). Physics and Applications of Microfluidics in Biology. Annu. Rev. Biomed. Eng..

[5] Sonker M., Sahore V., Woolley A.T. (2017). Recent advances in microfluidic sample preparation and separation techniques for molecular biomarker analysis: A critical review. Anal. Chim. Acta.

[6] Sonnen K.F., Merten C.A. (2019). Microfluidics as an Emerging Precision Tool in Developmental Biology. Dev. Cell.

[7] Saez J., Catalan-Carrio R., Owens R.M., Basabe-Desmonts L., Benito-Lopez F. (2021). Microfluidics and materials for smart water monitoring: A review. Anal. Chim. Acta.

[8] Marle L., Greenway G.M. (2005). Microfluidic devices for environmental monitoring. TrAC Trends Anal. Chem..

[9] Araz M.K., Tentori A.M., Herr A.E. (2013). Microfluidic Multiplexing in Bioanalyses. SLAS Technol..

[10] Li X., Zhou Y. (2021). Microfluidic Devices for Biomedical Applications.

[11] Sackmann E.K., Fulton A.L., Beebe D.J. (2014). The present and future role of microfluidics in biomedical research. Nature.

[12] Wu Q., Liu J., Wang X., Feng L., Wu J., Zhu X., Wen W., Gong X. (2020). Organ-on-a-chip: Recent breakthroughs and future prospects. BioMed. Eng. OnLine.

[13] Leung C.M., de Haan P., Ronaldson-Bouchard K., Kim G.-A., Ko J., Rho H.S., Chen Z., Habibovic P., Jeon N.L., Takayama S. (2022). A guide to the organ-on-a-chip. Nat. Rev. Methods Primers.

[14] Maged A., Abdelbaset R., Mahmoud A.A., Elkasabgy N.A. (2022). Merits and advances of microfluidics in the pharmaceutical field: Design technologies and future prospects. Drug Deliv..

[15] Kumar Thimmaraju M., Trivedi R., Hemalatha G., Thirupathy B., Mohathasim Billah A. (2023). Microfluidic revolution and its impact on pharmaceutical materials: A review. Mater. Today Proc..

[16] Gunes D.Z. (2018). Microfluidics for food science and engineering. Curr. Opin. Food Sci..

[17] Peng F., Ke Y., Zhao H., Tang Q., Zhang Z., Bai C. (2018). Application of microfluidic technology in oil industry—A new quick test method of fluid viscosity. IOP Conf. Ser. Mater. Sci. Eng..

[18] Lee W., Jerry F. (2007). Applied Biofluid Mechanics.

[19] Del Giudice F. (2022). A Review of Microfluidic Devices for Rheological Characterisation. Micromachines.

[20] Gupta S., Wang W.S., Vanapalli S.A. (2016). Microfluidic viscometers for shear rheology of complex fluids and biofluids. Biomicrofluidics.

[21] Mustafa A., Eser A., Aksu A.C., Kiraz A., Tanyeri M., Erten A., Yalcin O. (2020). A micropillar-based microfluidic viscometer for Newtonian and non-Newtonian fluids. Anal. Chim. Acta.

[22] Mustafa A., Haider D., Barua A., Tanyeri M., Erten A., Yalcin O. (2023). Machine learning based microfluidic sensing device for viscosity measurements. Sens. Diagn..

[23] Temam R. (2001). Navier-Stokes equations: Theory and Numerical Analysis.

[24] Temam R., Miranville A. (2005). Mathematical Modeling in Continuum Mechanics.

[25] Ariati R., Sales F., Souza A., Lima R.A., Ribeiro J. (2021). Polydimethylsiloxane Composites Characterization and Its Applications: A Review. Polymers.

[26] Seghir R., Arscott S. (2015). Extended PDMS stiffness range for flexible systems. Sens. Actuators A Phys..

[27] Guerrero B., Lambert M.F., Chin R.C. (2023). Transient behaviour of decelerating turbulent pipe flows. J. Fluid Mech..

[28] Urbanowicz K., Bergant A., Stosiak M., Deptuła A., Karpenko M. (2023). Navier-Stokes Solutions for Accelerating Pipe Flow—A Review of Analytical Models. Energies.

