# Supplementary material for: Optimizing Sensitivity in a Fluid-Structure Interaction-Based Microfluidic Viscometer: A Multiphysics Simulation Study
Source: Sensors (Basel). 2023 Nov 18;23(22):9265. doi: 10.3390/s23229265 (PMC10675072; doi:10.3390/s23229265)
Supplement: Supplementary file 1 [file sensors-23-09265-s001.zip › sensors-2614865-supplementary.pdf]

Supplementary Materials

S1: Simulation Methodology

The multiphysics simulations were conducted by fully coupling the laminar flow module with the solid mechanics module in COMSOL Multiphysics, utilizing a fluid-structure interaction (FSI) sub-module. These simulations employed a user-controlled mesh. Below, we provide details of the simulation parameters and mesh specifications in Table S1:

Table S1: Simulation Methodology.

Modules	Boundary Conditions and Simulation Parameters
**Laminar flow**	**Inlet:** Fully developed flow & flow rate.<br>**Outlet:** Static pressure & $P = P_a$ (atmospheric pressure)<br>Outlet pressure set to zero ($P = 0$), with the pressure at the physical interface set to atmospheric pressure (P_a).<br>**Wall:** No slip
**Solid mechanics**	**Linear elastic material:** Isotropic material, Young's modulus, and Poisson ratio. **Young's modulus:** 1.6 MPa, **Poisson Ratio:** 0.49<br>**Fixed constraint:** The bottom of micropillars was a fixed constraint.
**Fluid-structure interaction**	**Fluid:** Laminar flow<br>**Structure:** Solid mechanics<br>**Fixed Geometry coupling:** Fully Coupled
**Mesh**	**User-controlled:** General Physics (free tetrahedral & boundary layers)<br>**Max element size:** 700<br>**Min element size:** 126<br>**Max element growth:** 1.8

We systematically tested three distinct mesh sizes - Coarse Mesh, Normal Mesh, and Fine Mesh. The objective behind this was to ensure that our simulations could precisely represent the fluid-structure interactions while optimizing computational efficiency. The key specifications for these mesh sizes are as follows:

- Coarse Mesh: Maximum element size: 1330 µm, Minimum element size: 180 µm
- Normal Mesh: Maximum element size: 700 µm, Minimum element size: 126 µm
- Fine Mesh: Maximum element size: 560 µm, Minimum element size: 70 µm

Figure S1 presents the impact of mesh size on simulation results, specifically micropillar tip displacement as a function of viscosity for the three different mesh sizes. Our observations revealed that an increase in mesh size only marginally changed the simulation results. Shifting from Coarse mesh to Normal mesh, for instance, raised the displacement by a mere 2.7% at both low and high flow rates. Similarly, transitioning from Coarse to Fine mesh improved results by only 4.7%. While the

precise cause of the marginal rise in micropillar displacement with larger mesh sizes remains unclear, it might be due to a blend of factors including the geometric resolution of micropillars, numerical artifacts, interpolation inaccuracies, and sensitivity to boundary conditions.

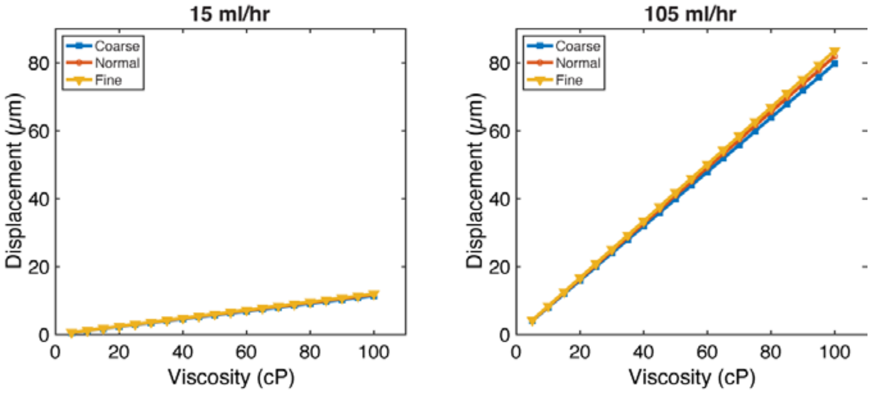

Figure S1: Impact of mesh size on simulation results. Micropillar tip displacement as a function viscosity for three different mesh sizes. The results indicate that increasing the mesh size results in a marginal change in displacement. As a result, we picked the "normal" mesh size (Maximum element size: 700 μm, Minimum element size: 126 μm) to maintain the balance between computational speed and accuracy for the simulations conducted in this work.

As a result, all simulations in this work were performed using the "Normal mesh" setting (Maximum element size: 700 μm, Minimum element size: 126 μm), offering a balanced approach to computational resources and simulation accuracy. This configuration effectively captures the fluid-structure interactions within the microchannel while preserving computational efficiency, striking a favorable trade-off between simulation speed and precision.

S2: Prior experimental validation of the simulation model

Simulation results presented in this work are based on a design framework established in our earlier studies (Main text, references [21-22]), where we developed and experimentally validated a multiphysics model of the microfluidic viscometer. This earlier study demonstrated a robust correlation between simulation results from the multiphysics model and experimental data, providing the basis for the simulations presented in this manuscript.

In our prior work, we compared results from experiments and multiphysics simulations to validate our multiphysics model of the microfluidic viscometer. To match simulation parameters with microfluidics experiments, we set $D = 300$ μm, $H = 1500$ μm, corresponding to a micropillar aspect ratio of 5:1, $g = 100$ μm, $d = 400$ μm, and $CW = 900$ μm. The simulations were conducted to determine micropillar tip displacement as a function of flow rate (15–105 ml/hr) across multiple viscosity values (5–50 cP). Figure S2 illustrates the comparison between experimental and multiphysics simulation data, showing good agreement at low viscosity values across all flow rates. At higher viscosity values, a strong agreement is observed particularly at low and medium flow rates.

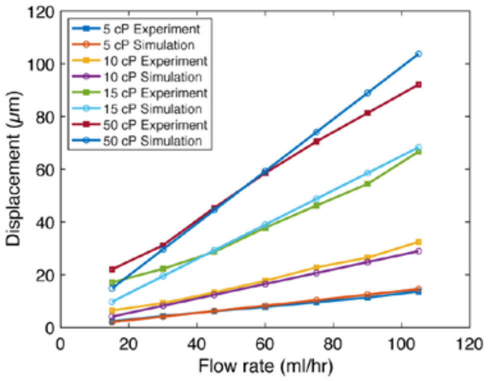

Figure S2: Experimental validation of the multiphysics model for the microfluidic viscometer. Micropillar tip displacement as a function of flow rate (15–105 ml/hr) for various viscosity values (5–50 cP). The results indicate a notable agreement at low viscosity values across all flow rates. Furthermore, for higher viscosity values, the congruence between experimental and simulation results is robust, particularly in the low to middle range of flow rates.

Overall, these results underscore the reliability and accuracy of our multiphysics modelling of the microfluidic viscometer, demonstrating consistency with experimental results. Bolstered by this validation, we leveraged these multiphysics simulations to explore the influence of geometric device parameters—such as micropillar dimensions, aspect ratio, pillar spacing, and the distance between pillars and channel walls—on the sensitivity of the microfluidic viscometer.

S3: Effect of gap (g) between the micropillar tip and channel ceiling on viscometer sensitivity for three different aspect ratios (AR)

We carried out simulations to explore the impact of the gap between the micropillar tip and channel ceiling on viscometer sensitivity. Figure S3 depicts the relationship between pillar displacement and viscosity at various g values for micropillar arrays with $AR = 3{:}1$, whereas Figures S4 and S5 show the same plots for $AR = 4{:}1$ and $AR = 5{:}1$, respectively. From the slope of these curves, the sensitivity data, as presented in Figure 3 in the main text, are deduced. Notably, alterations in the gap initially yield slight increase in the slope of the pillar displacement vs. viscosity curves followed by a significant decrease, indicating that there is an optimal gap for maximum sensitivity.

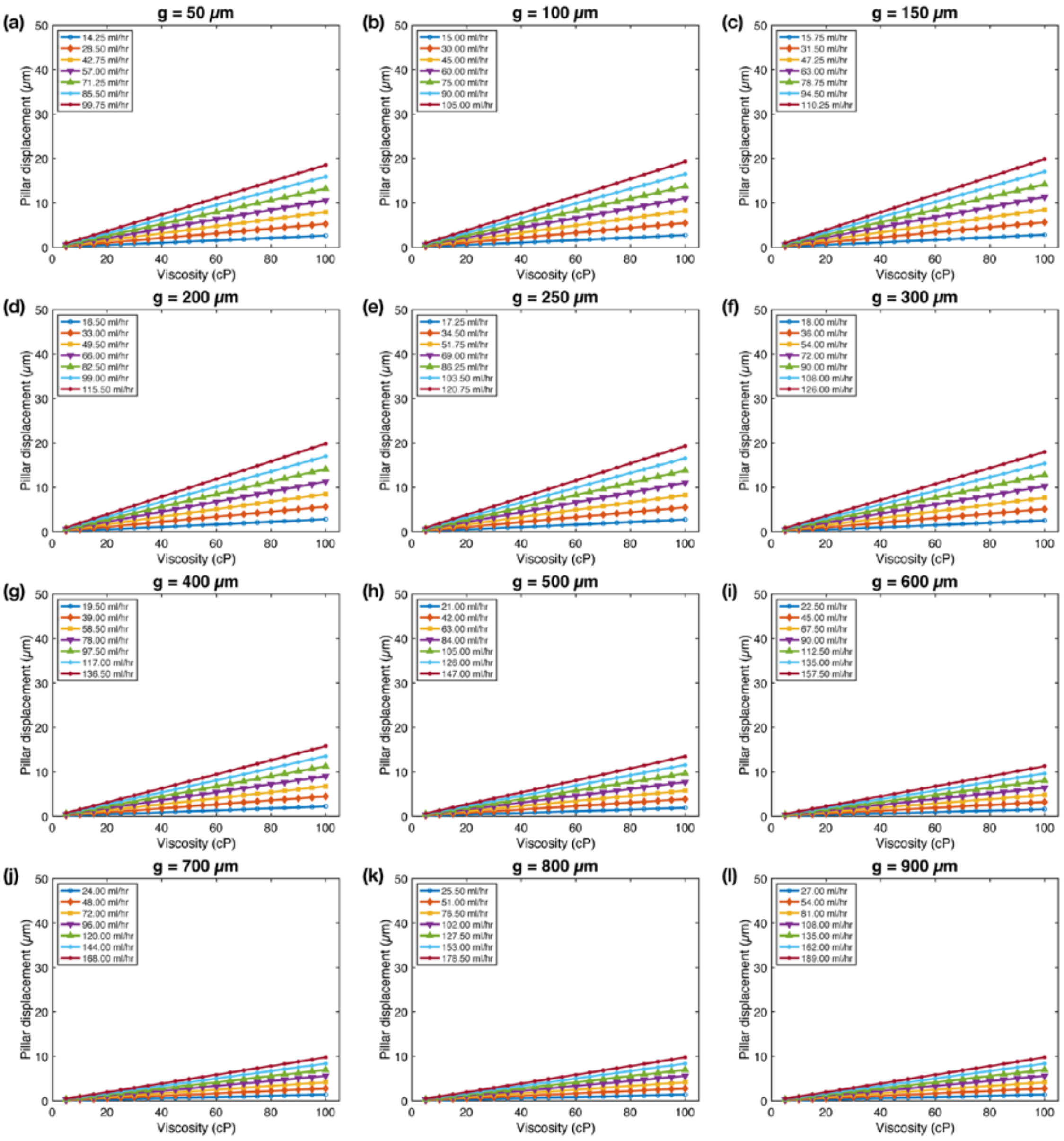

Figure S3: Investigating the impact of gap (g) on viscometer sensitivity Pillar displacement vs. viscosity plots at various g values for micropillars with $AR = 3{:}1$. Interestingly, the viscometer

sensitivity exhibits an initial rise followed by a decline with increasing g, as evidenced by the slope of the pillar displacement vs. viscosity plots.

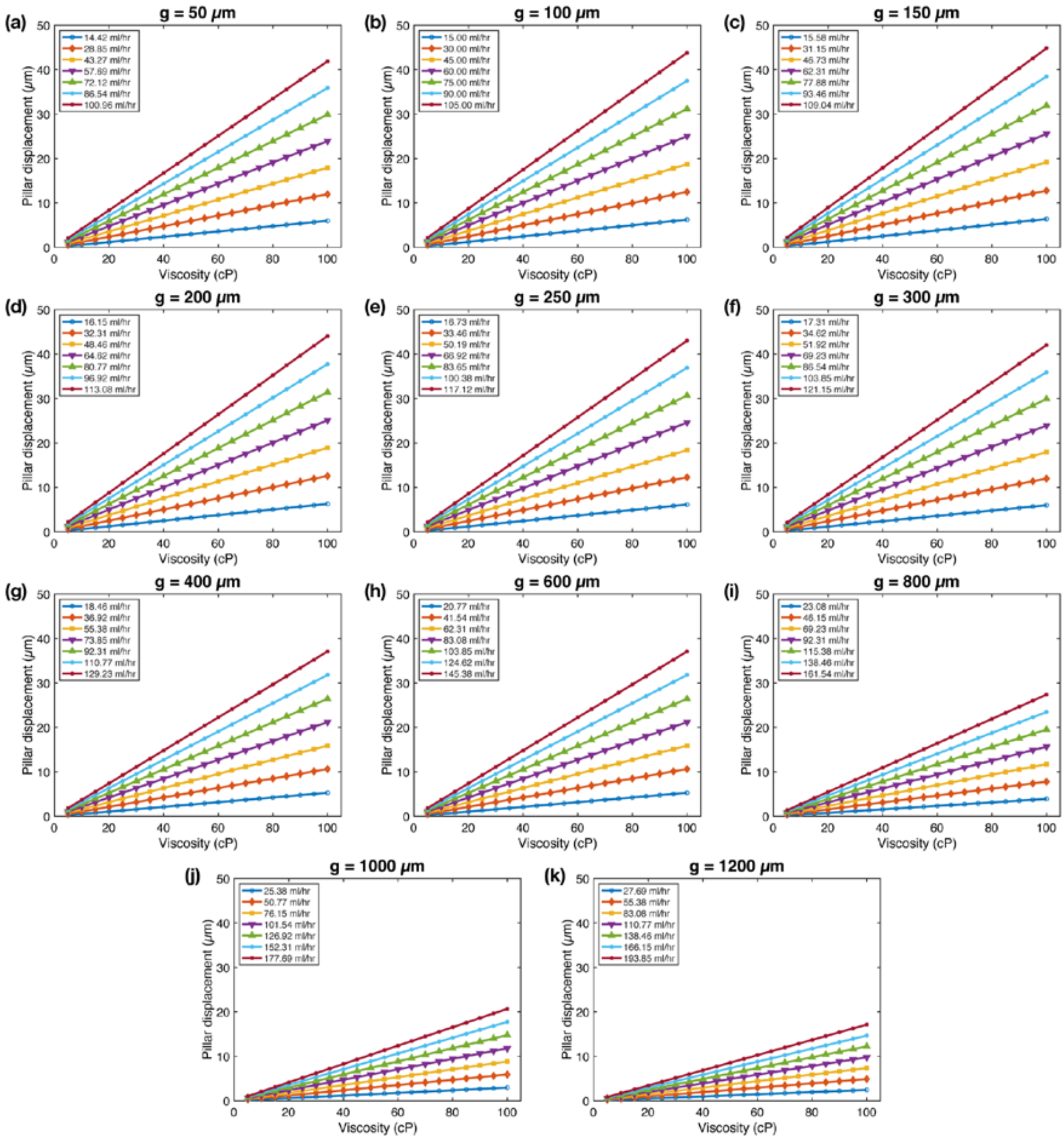

Figure S4: Investigating the impact of gap (g) on viscometer sensitivity Pillar displacement vs. viscosity plots at various g values for micropillars with $AR = 4:1$.

Figure S5(a-b) illustrates the fluid velocity distribution across the microchannel, shown at a mid-channel cross section, for a micropillar array with $AR = 5:1$. In Figure S5a, corresponding to $g =$ 50 μm (with $H = 1500$ μm and $CH = 1550$ μm), we observe that the fluid velocity attains its maximum value around the micropillar array, indicating increased fluid-micropillar interactions. In contrast, Figure S5b, corresponding to $g = 300$ μm (with $H = 1500$ μm and $CH = 1800$ μm), illustrates that the fluid velocity reaches its peak value within the gap region, implying a reduction in fluid-micropillar interactions.

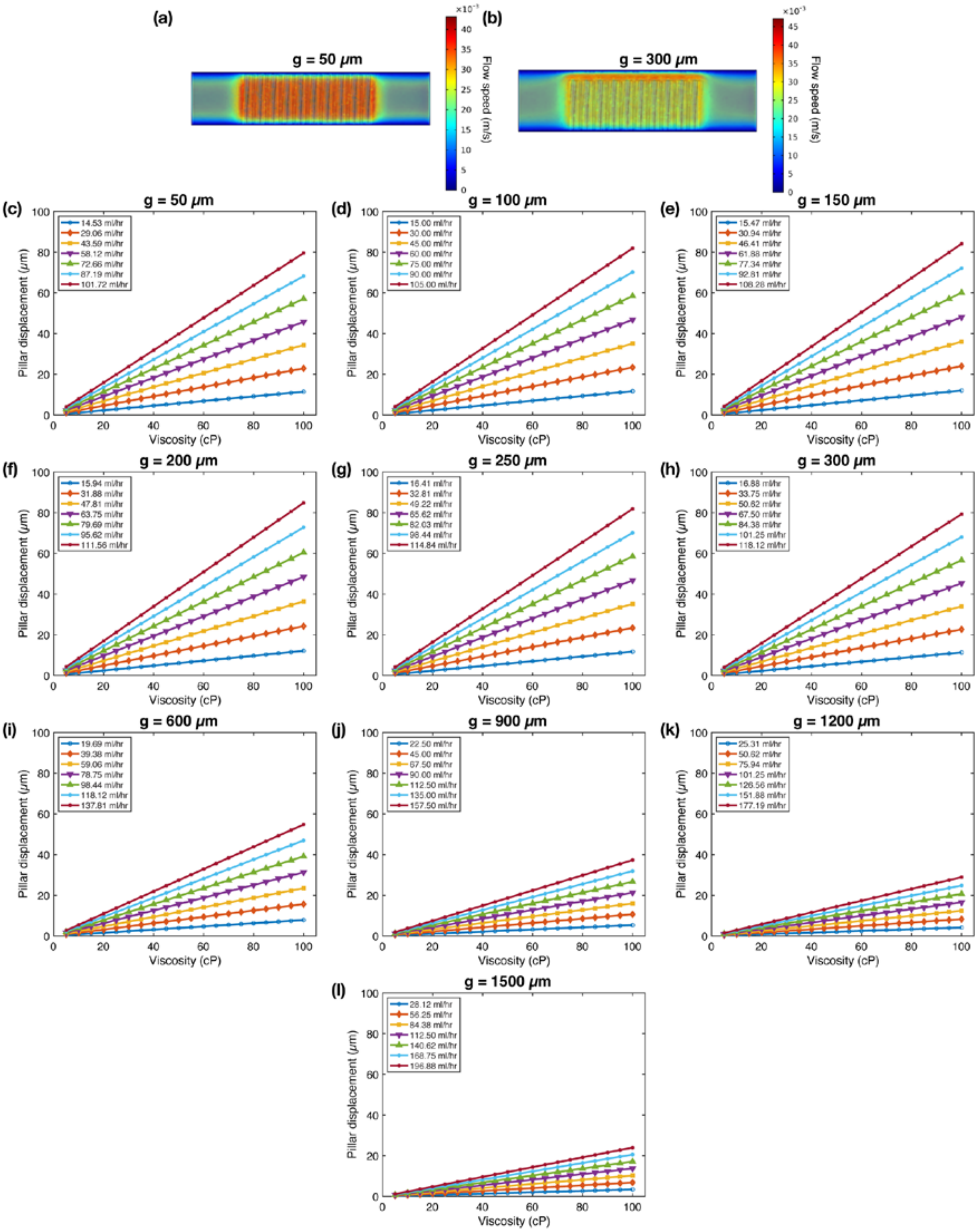

Figure S5: Investigating the impact of gap (g) on viscometer sensitivity. (a-b) Visualization of fluid velocity distribution across the channel length at a mid-channel cross section for a micropillar array with $AR = 5{:}1$. The widening gap, transitioning from $g = 50$ μm to $g = 300$ μm, corresponds to a decrease in fluid velocity surrounding the micropillar array and a simultaneous increase within the gap region, suggesting a reduction in fluid-micropillar interactions. **(c-h)** Pillar displacement vs. viscosity plots at various g values for micropillars with $AR = 5{:}1$.

S4: Effect of channel width (CW) on viscometer sensitivity for three different aspect ratios (AR)

We conducted simulations to explore the impact of the channel width on viscometer sensitivity. Figures S6-S8 depict the relationship between pillar displacement and viscosity for different channel widths, spanning from $CW = 700 - 900$ µm, for viscometers with $AR = 3:1$, $AR = 4:1$ and $AR = 5:1$, respectively. The sensitivity data presented in Figure 4 in the main text are deduced from the slopes of these plots. Notably, as the channel width increases, the slopes of the pillar displacement vs. viscosity curves decrease, implying that the sensitivity of the viscometer diminishes as CW increases.

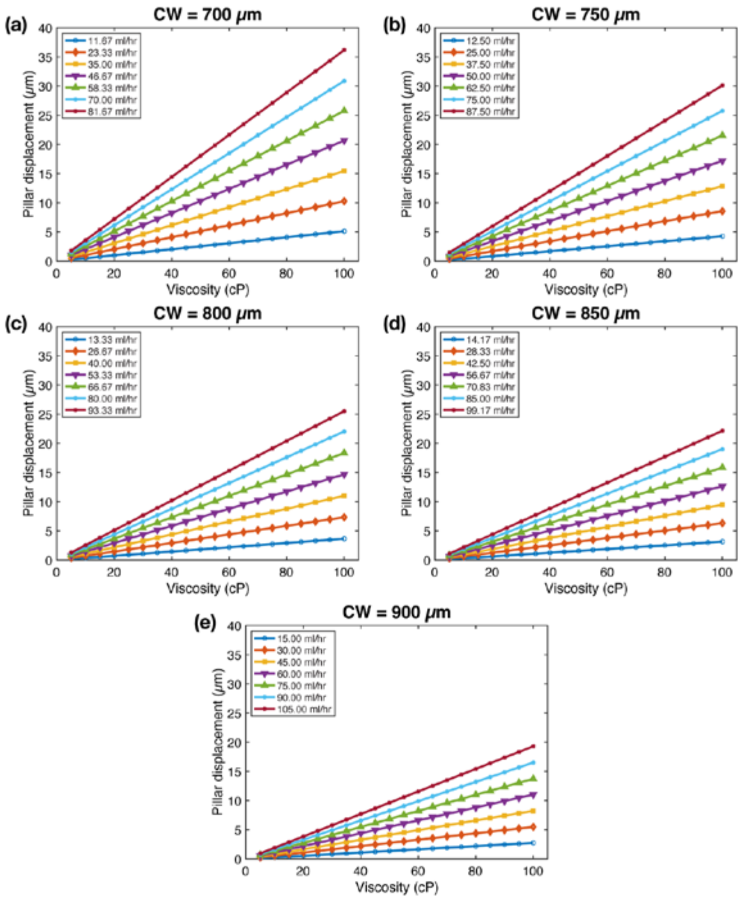

Figure S6: Investigating the impact of channel width (CW) on viscometer sensitivity for micropillars with $AR = 3:1$. As the channel widens from $CW = 700\ \mu m$ to $CW = 900\ \mu m$, the slope of pillar displacement vs. viscosity plots decreases, indicating a corresponding decline in sensitivity as the channel width increases.

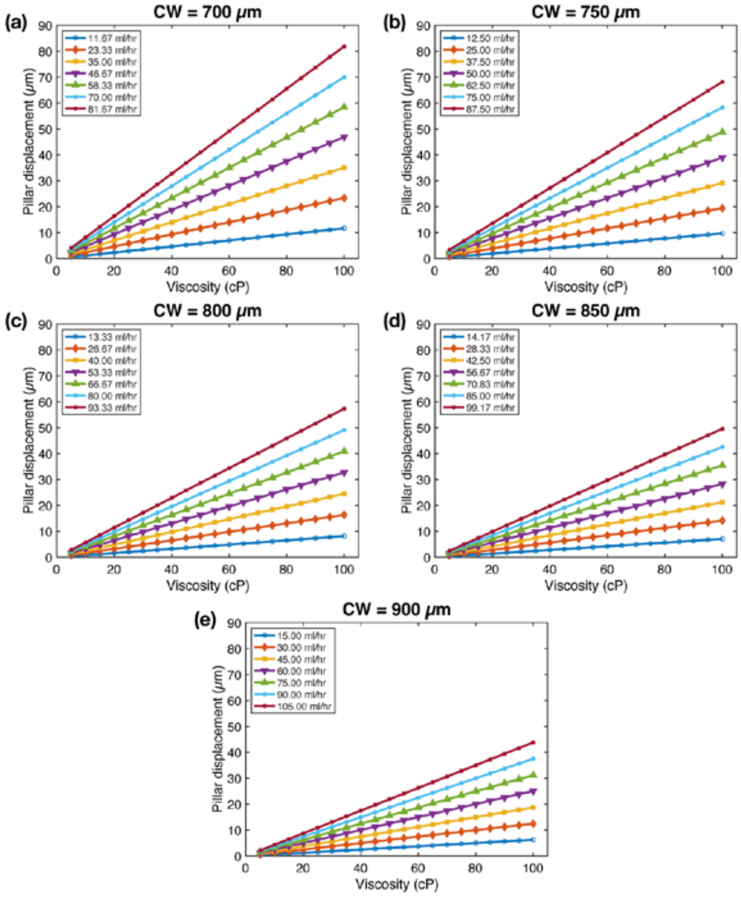

Figure S7: Investigating the impact of channel width (CW) on viscometer sensitivity for micropillars with $AR = 4:1$. Pillar displacement vs. viscosity plots at various channel widths for micropillars with $AR = 4:1$.

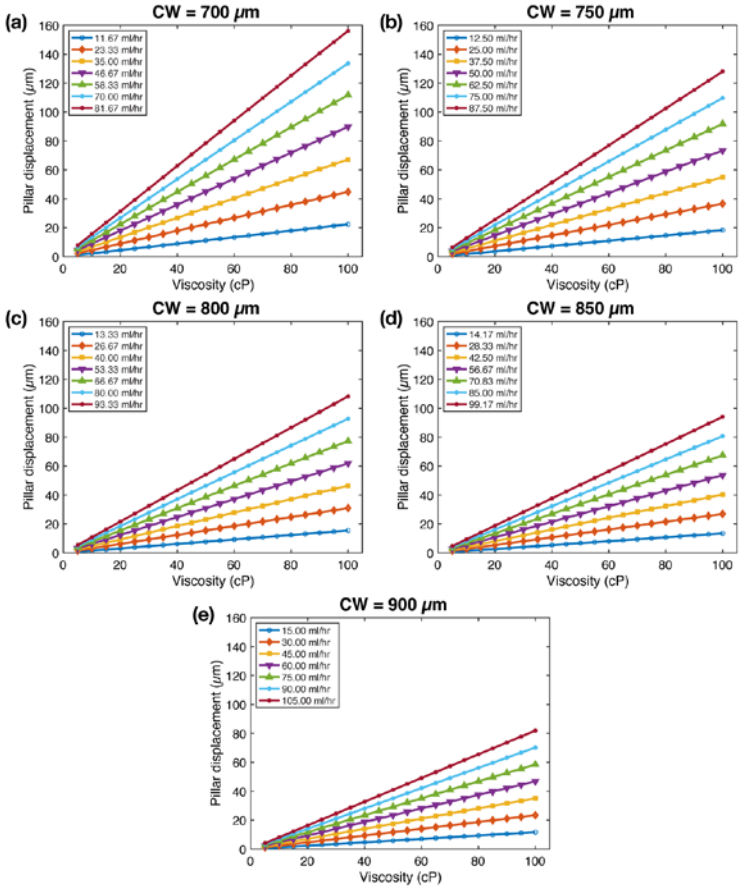

Figure S8: Investigating the impact of channel width (CW) on viscometer sensitivity for micropillars with $\boldsymbol{AR = 5:1}$. Pillar displacement vs. viscosity plots at various channel widths for micropillars with $AR = 5:1$.

S5: Effect of pillar spacing (d) on viscometer sensitivity

We carried out simulations to analyze the influence of the pillar spacing on viscometer sensitivity. Figure S9 illustrates the relationship between pillar displacements and viscosity for six different pillar spacings, ranging from $d = 350 - 600$ μm. As the pillar spacing increases, there is a modest increase in the slopes of the pillar displacement vs. viscosity curves, indicating enhanced viscometer sensitivity. The sensitivity data presented in Figure 5 in the main text are calculated using the slopes of the curves in Figure S9.

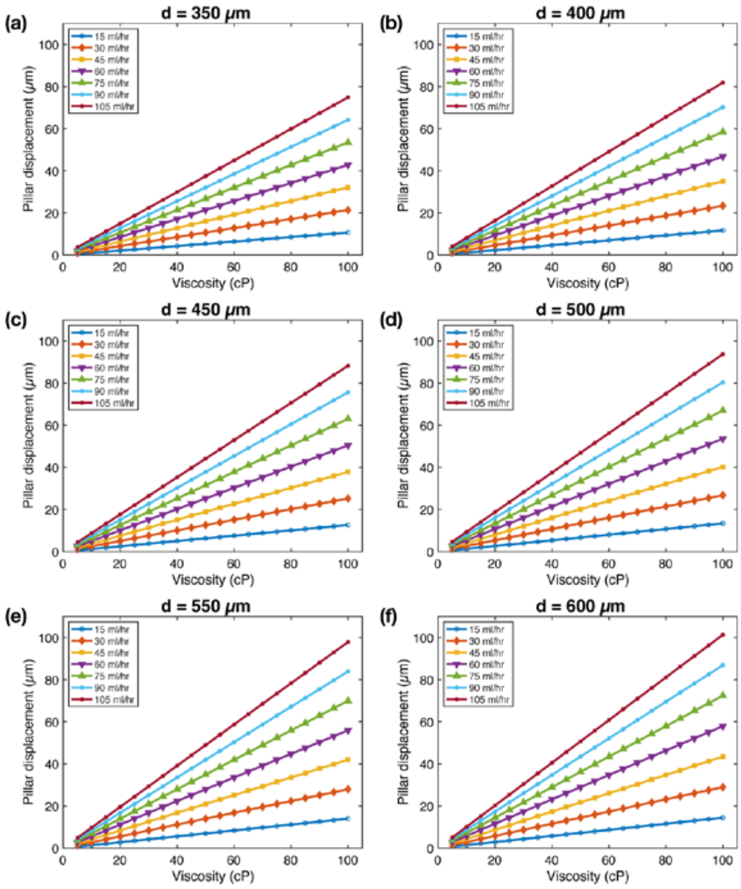

Figure S9: Investigating the impact of pillar spacing (d) on viscometer sensitivity for micropillars with $AR = 5:1$. As the pillar spacing is increased from $d = 350$ μm to $d = 600$ μm, there is a modest increase in the slope of pillar displacement vs. viscosity plots, indicating a concomitant improvement in sensitivity attributed to variation in pillar spacing.

S6: Effect of Young's modulus (E) on viscometer sensitivity

We performed simulations to analyze the influence of the Young's modulus of the micropillars on viscometer sensitivity. Figure S10 illustrates the relationship between pillar displacements and viscosity for seven different Young's moduli, ranging from $E = 1.3 - 3.1$ MPa, reflecting the materials properties of PDMS micropillars. Increasing Young's modulus leads to a reduction in the slopes of the pillar displacement vs. viscosity curves, implying a decrease in viscometer sensitivity. The sensitivity data presented in Figure 6 in the main text are calculated using the slopes of the curves in Figure S10.

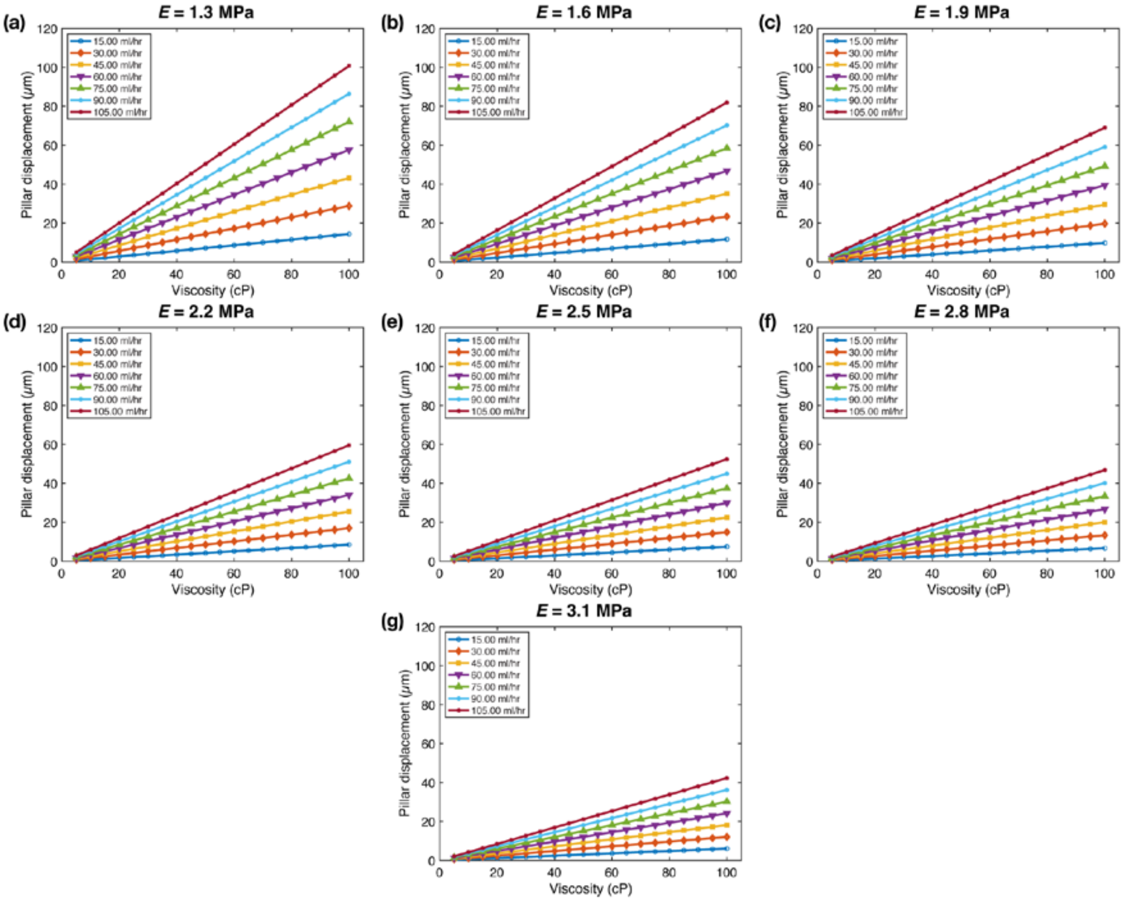

Figure S10: Investigating the impact of Young's modulus (E) on viscometer sensitivity for micropillars with $AR = 5{:}1$. As the Young's modulus is increased from $E = 1.3$ MPa to $E = 3.1$ MPa, there is a substantial decrease in the slope of pillar displacement vs. viscosity plots, indicating a decline in sensitivity associated with changes in Young's modulus.
